# Machine-learning algorithms for asthma, COPD, and lung cancer risk assessment using circulating microbial extracellular vesicle data and their application to assess dietary effects

**DOI:** 10.1038/s12276-022-00846-5

**Published:** 2022-09-30

**Authors:** Andrea McDowell, Juwon Kang, Jinho Yang, Jihee Jung, Yeon-Mok Oh, Sung-Min Kym, Tae-Seop Shin, Tae-Bum Kim, Young-Koo Jee, Yoon-Keun Kim

**Affiliations:** 1 Institute of MD Healthcare, Inc, Seoul, Republic of Korea; 2 Department of Pulmonary and Critical Care Medicine, and Clinical Research Center for Chronic Obstructive Airway Disease, Asan Medical Center, Seoul, Korea; 3 Department of Internal Medicine, Inje University Haeundae Paik Hospital, Inje University College of Medicine, Busan, Republic of Korea; 4 Department of Allergy and Clinical Immunology, Asan Medical Center, University of Ulsan College of Medicine, Seoul, Korea; 5 Department of Internal Medicine, Dankook University College of Medicine, Cheonan, Korea

**Keywords:** Respiratory tract diseases, Machine learning, Predictive markers

## Abstract

Although mounting evidence suggests that the microbiome has a tremendous influence on intractable disease, the relationship between circulating microbial extracellular vesicles (EVs) and respiratory disease remains unexplored. Here, we developed predictive diagnostic models for COPD, asthma, and lung cancer by applying machine learning to microbial EV metagenomes isolated from patient serum and coded by their accumulated taxonomic hierarchy. All models demonstrated high predictive strength with mean AUC values ranging from 0.93 to 0.99 with various important features at the genus and phylum levels. Application of the clinical models in mice showed that various foods reduced high-fat diet-associated asthma and lung cancer risk, while COPD was minimally affected. In conclusion, this study offers a novel methodology for respiratory disease prediction and highlights the utility of serum microbial EVs as data-rich features for noninvasive diagnosis.

## Respiratory disease: Microbial eavesdropping aids diagnosis

Artificial intelligence (AI) has enabled researchers to intercept microbial messages bearing clinically useful information about of a variety of respiratory disorders. The organisms that comprise our microbiome communicate via the release of tiny, biomolecule-laden membrane bubbles called ‘extracellular vesicles’ (EVs) into the bloodstream. EVs are also influenced by human disease. South Korean researchers led by Yoon-Keun Kim of MD Healthcare, Seoul, and Young-Koo Jee of Dankook University College of Medicine, Cheonan, have used an AI algorithm to assemble EV-based profiles that can discriminate between healthy people and those with conditions like asthma or lung cancer. Their analysis of 1727 patient serum samples revealed microbial signatures that enabled accurate diagnosis of several respiratory disorders. Preliminary experiments in mice suggest that certain dietary changes could help shift the microbiome of high-risk individuals towards a healthier profile.

## Introduction

The incidence of chronic obstructive pulmonary disease (COPD) has been steadily increasing, and the disease accounted for 3.0 million deaths in 2016, becoming the third most common global cause of death according to the World Health Organization^1^. COPD is a particularly urgent public health threat in countries such as South Korea with long-term exposure to airborne particulate matter and a high prevalence of smoking, especially among men^2^. Asthma is one of the most common noncommunicable diseases in the world, and although less deadly than COPD, asthma contributed 24.8 million DALYs (disability adjusted life years) in 2016^3^. Chronic respiratory tract inflammation can induce carcinogenic activity, such as DNA damage, mutations, inactivation of tumor suppressor genes, and apoptosis evasion, which leads to increased lung cancer risk^4–6^. Therefore, it is imperative to establish accurate predictive methods for COPD, asthma, and lung cancer for the early treatment, prevention and reduction of the respiratory disease burden.

The human microbiome accounts for over 99% of the genomic material in our bodies, and microbial protein-encoding genes are 360 times more abundant than human protein-encoding genes^7^. Massive global efforts have been launched in the past decade to understand the underlying relationship between our bacterial counterparts and human health. Mounting evidence supports the tremendous influence our microbiome has on health and disease, with roles in metabolism, immune modulation, and pathogen protection among other functions^8,9^. However, dysbiotic microbiota composition and activity have been associated with a variety of diseases, including metabolic syndrome, immune disorder, and cancer^10–12^.

Recently, interest has shifted from assessing the composition of the microbiome to understanding the functional roles it plays in human health. An emerging functional component of the microbiome is the role of microbial extracellular vesicles (EVs) in systemic microbiome activity. As awareness of the significant role EVs play in interkingdom intercellular transport and communication has risen, recent attention has focused on the influence of microbial EVs on health and disease. Microbial EVs are nanosized vesicles approximately 100 nm in diameter that are involved in cell-to-cell communication and deliver microbial components, including DNA, RNA, proteins, and lipids, throughout the body^13^.

The influential role of microbial EVs on human health has become increasingly apparent over the past decade, with recent research highlighting the differentially protective and harmful effects of microbial EVs on health. For example, while inhalation of *Staphylococcus aureus* EVs has been shown to induce neutrophilic pulmonary inflammation^14^, systemic injection of various bacterial EVs was reported to induce a powerful antitumour response in the lungs of mice injected with metastatic carcinoma and melanoma cells^15^. Furthermore, metagenomic analysis of bacterial EVs has revealed differential proportions of bacterial EVs between a variety of diseases and healthy controls^16–18^. As microbial EVs can enter the circulation through the epithelial cells lining the gut and other barriers that bacteria are unable to bypass, circulating microbial EVs offer an ideal diagnostic target to holistically monitor systemic microbiome activity.

Here, we sought to develop novel predictive models for COPD, asthma, and lung cancer risk assessment through artificial intelligence (AI) modeling of respiratory patient-derived serum microbial EV metagenomic data. Furthermore, we tested these diagnostic models in mice to determine any dietary supplements capable of reducing high-fat diet (HFD)-associated COPD, asthma, and lung cancer risk. The results of this study establish the strong potential of serum microbial EVs as an accurate, noninvasive data source for respiratory disease diagnosis.

## Materials and methods

### Subjects and serum sample collection

A total of 1825 Korean COPD patients (*n* = 93), asthma patients (*n* = 454), lung cancer patients (*n* = 283), and healthy control subjects (*n* = 995) were enrolled from Konkuk University Medical Center (Seoul), Asan Medical Center (Seoul), Samsung Medical Center (Seoul), Dankook University Hospital (Cheonan) and Inje University Haeundae Paik Hospital (Busan) from 2017 to 2020. Each COPD and asthma clinical subject showed severe COPD or asthma symptoms, respectively, leading them to visit the Konkuk University Medical Center or Asan Medical Center for treatment. Lung cancer patients enrolled from Dankook University Hospital and Samsung Medical Center were verified to be currently diagnosed with lung cancer. Control subjects were screened through a general health examination at Haeundae Paik Hospital. The present study was approved by the Institutional Review Boards of Asan Medical Center (IRB No. 2014-0360), Dankook University Hospital (IRB No. 2014-01-002-016), Inje University Haeundae Paik Hospital (IRB No. 129792-2015-064), Konkuk University Medical Center (IRB No. KUH1010338), and Samsung Medical Center (IRB No. 2013-10-112-001 and 2018-03-130-001). All methods were conducted in accordance with the approved guidelines, and informed consent was obtained from all clinical subjects.

### Microbial EV DNA extraction and sequencing

All collected human serum samples were transferred to serum separator tubes (SSTs), and EVs in the serum were extracted using a DNeasy Blood & Tissue Kit (QIAGEN, Germany) after centrifugation, filtering, and boiling as previously described^19^. The extracted EV DNA in each sample was quantified using QIAxpert (QIAGEN, Germany). Isolated microbial genomic DNA was amplified as previously described by targeting the V3-V4 hypervariable regions of the 16S rDNA gene^19^. The libraries were prepared using PCR products and quantified using a QIAxpert system (QIAGEN, Germany). After quantification, all amplicons were sequenced using a MiSeq (Illumina, USA) instrument.

### Taxonomic assignment of microbial EVs

Taxonomic assignment was performed by the profiling program MDx-Pro ver. 2 (MD Healthcare, Korea). Briefly, paired-end reads were trimmed and merged using Cutadapt version 1.1.6 and CASPER, respectively. High-quality sequencing reads were obtained by discarding sequences with read lengths below 350 and above 550 bp and with Phred quality scores below 20. Operational taxonomic units (OTUs) were clustered using the VSEARCH de novo clustering method with a 97% similarity threshold. Subsequently, taxonomic assignment was conducted against the Silva 132 sequence database under default parameters. If clusters could not be assigned at the genus level due to insufficient taxonomic information in the database, the taxon was assigned at the next highest level, as indicated in parentheses. Brackets around the taxon name represent an unverified, suggested taxonomic assignment based primarily on whole-genome phylogeny. Finally, samples were filtered to remove those with fewer than 1000 OTUs for use in downstream analysis.

### Data coding through taxonomic hierarchal accumulation

Prior to use as features for the AI models, we employed a novel taxonomic hierarchal accumulation method to give weight to imprecisely classified genera through their verified higher-level taxonomy while simultaneously reducing the influence of zero-inflation in the microbiome relative abundance data. First, assigned species were summarized to their respective genus levels, and all abundance values were scaled by log2. Next, the sum of taxa abundance values in each sample was set to one to represent the relative abundance of each OTU. Finally, the taxonomic values from the genus to kingdom levels were accumulated from all samples using the following formula:

*V*_ACCUMULATION_ = *V*_G_ + (*k*_1_*V_F_) + (*k*_2_*V_O_) + (*k*_3_*V_C_) + (*k*_4_*V_P_) + (*k*_5_*V_K_)

*V*_ACCUMULATION_: Accumulated value of a genus

*V*_G_: Relative abundance of a genus

*V*_O_/_C_/_P_/_K_: Sum of *V*_G_ for genus’s higher taxonomy (order, class, phylum, kingdom)

*k*_i_ = 10^–(1 + *i*)^

The accumulated taxonomic values of the sequences obtained from the microbial EVs in all patient serum samples were calculated by the above formula and used for downstream disease-predictive analysis.

### Machine-learning (ML) algorithm for the prediction of respiratory diseases

After sample filtering, 1513 serum microbial EV taxa were selected as features for respiratory disease model development. Five ML methods were applied to the control and disease group samples for asthma, COPD, and lung cancer: a generalized linear model (GLM) without feature selection, a GLM with feature selection, a gradient boosting machine (GBM), an artificial neural network (ANN), and a GBM + ANN ensemble model. The GLM without feature selection incorporated all 1513 serum microbial EV taxa features in the model and was created using the stats basic package in R (version 3.6.3). The GLM with feature selection implemented a Wilcoxon test to filter out features with Bonferroni adjusted p values lower than 0.05. For the GBM, we set the GradientBoostingRegressor of the scikit-learn package (version 0.21.3) to learning_rate = 0.01, n_estimators = 3000, max_depth = 10. For the ANN model, the loss function = mean square error (MSE) function, activation function = relu, optimizer = RMSProp, and epoch = 200 were set using the Keras package, to which the 5-layer modeling algorithm applied with L1 regularization was applied. All 1513 data features were incorporated in the GBM and ANN methods, and the ensemble method incorporated the average value of outputs from the GBM and ANN methods. To overcome the limitations caused by overfitting, cross-validation was applied with the data randomly split into train-test sets at a 7:3 ratio for 30 iterations. The training datasets were used for model training, and the test sets were used for validation. Finally, feature importance was determined by implementing permutative feature importance analysis for 30 iterations using the eli5 package (version 0.10.1) in Python.

### In vivo animal study

A total of 180 female C57BL/6 mice were obtained from OrientBio Korea (Sungnam, Korea) at the age of 6 weeks. Mice were housed at a constant temperature (22° ± 2°), humidity (50% ± 5%), and photoperiod (08:00 to 20:00) with 5 mice per individually ventilated cage (IVC). After the allowance of 1 week for the mice to adjust to their new environment, each cage was randomly assigned to one of 36 dietary groups: normal chow diet (NCD), HFD, and HFD plus lotus root flower, sesame oil, balloon flower root extract, rich soybean paste powder, mungbean powder, glutinous rice flour, rice grain syrup, black garlic extract, Korean pancake powder, colovita, Rubus schizostylus Leveille, Safflower extract, onion extract, Kakadu plum powder, brown rice oil, ginger powder, pear extract, rice powder, propolis spray, MSG, black herbal tea, propolis, honey, sorghum powder, acacia honey, oriental raisin nut extract, red ginseng extract, buckwheat powder, kelp powder, shark cartilage calcium, shiitake powder, turmeric powder, mealworm oil, and policosanol. The NCD consisted of normal laboratory rodent chow (Cat. No. 38057; Purina, Seongnam, Korea). The HFD feed was obtained from Research Diets (Cat. No. D12492; New Brunswick, NJ, USA) and was composed of 60% fat, 20% protein, and 20% carbohydrates. Supplemental functional foods were all purchased from the local market and added to the drinking water at a 2% ratio (2 g per 100 mL of water) and alternated with regular drinking water every 12 h. After 4 weeks of the HFD or HFD + Supplement dietary conditions, mice were sacrificed, and 0.5 mL of serum was collected from the heart. Mouse serum samples were processed as described above for NGS sequencing of circulating EVs, and respiratory disease prediction values for asthma, COPD, and lung cancer were calculated based on the ensemble algorithms developed in this study. All animal experiments were approved by the Review Board of Chung-Ang University (Approval No. 2018-00057).

### Statistical analysis

The alpha diversity of the variance within each clinical sample was assessed using the a-diversity test in the phyloseq package in R for the total observed OTUs, richness estimates Chao1 and ACE (abundance-based coverage estimator), and the Shannon and Simpson diversity indices. Dimension reduction was conducted to assess the beta diversity between clinical samples based on the UniFrac distance using principal component analysis (PCA) and multiple dimension scale (MDS) in the stats package in R. Additionally, t-distributed stochastic neighbor embedding (t-SNE), a nonlinear machine-learning technique for visualization of high-dimensional data, was conducted using the tsne package in R. The permutation feature importance was determined for each coded feature using the ELI5 package in Python 3.6. Significant differences between groups were determined by either Pearson’s correlation, *t*-test, or Wilcoxon test with significance established at *p* ≤ 0.05.

## Results

### Demographic characteristics of the study subjects

Of the 1821 samples collected, a total of 1727 samples met the criteria for downstream analysis as described above, including 92 COPD patients, 428 asthma patients, 279 lung cancer patients, and 928 healthy control subjects (Supplementary Table 1). The mean ages of the four groups ranged from 51.53 to 69.68 years with an SD of 7.33 to 15.59 years. The sex ratio of the study participants varied widely among the four groups. The control and asthma groups were skewed toward female subjects with male-to-female sex ratios of 0.35 and 0.69, respectively. Meanwhile, male COPD samples were dramatically higher in number than female COPD samples, with a male-to-female ratio of 22.00, and lung cancer skewed males at a lower ratio of 1.68. Despite these disparities, no significant correlation was determined between either age or sex and the serum microbial EV samples (Supplementary Fig. 1).

### 16S rDNA metagenomic sequence data assessment

**Fig. 1:**
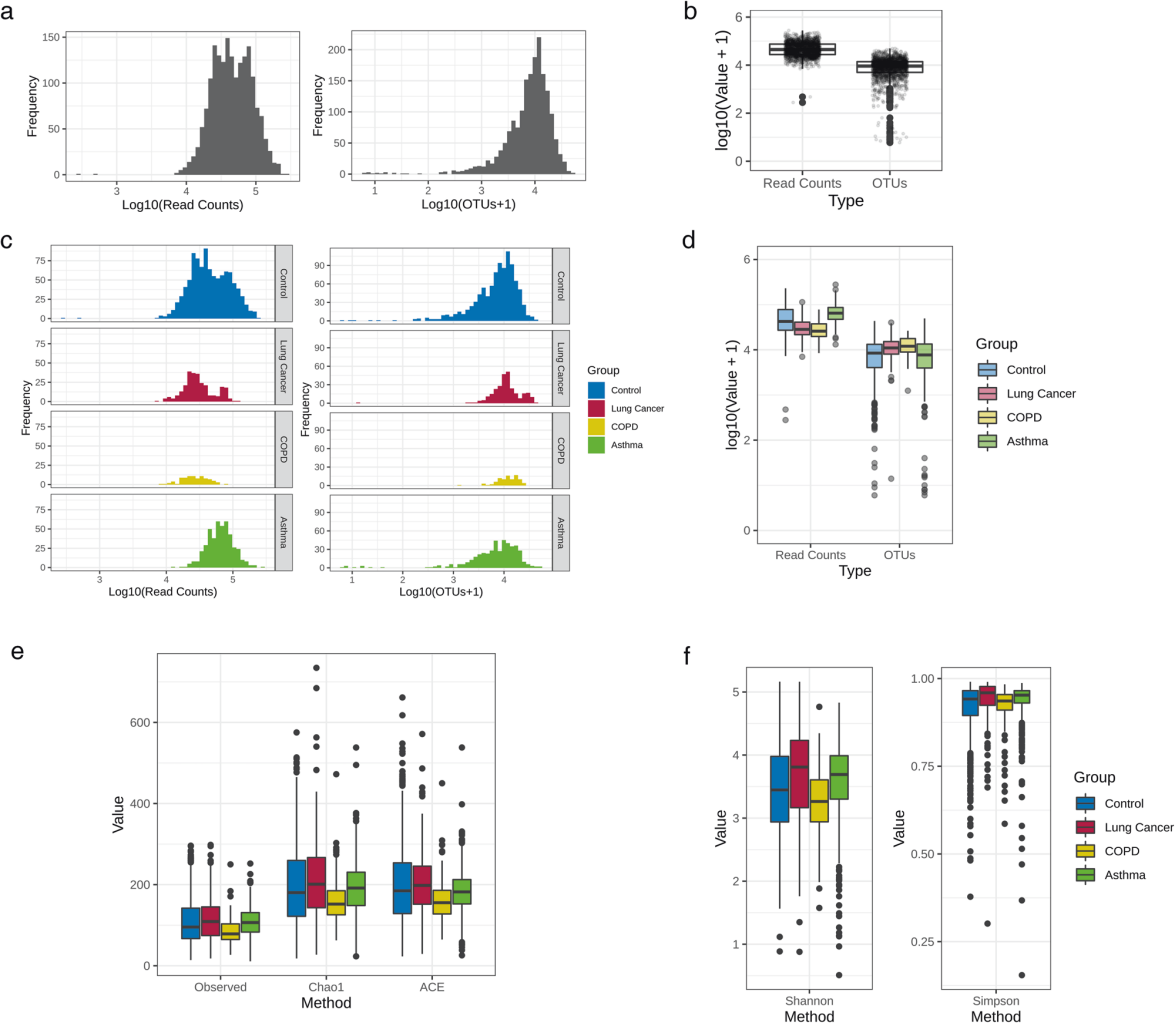
Raw NGS read counts, profiled OTU counts, and alpha diversity of serum clinical samples. Frequency distribution of **a** all clinical serum samples and **b** the samples in the control, lung cancer, COPD, and asthma groups based on their read counts and number of OTUs. A box-plot was also constructed to visualize the read counts and number of OTUs in **c** all samples and **d** the individual clinical groups. Alpha diversity was assessed between the four clinical groups through **e** species richness (observed, Chao1, and ACE), and **f** species diversity (Shannon and Simpson indices) within each sample in a given group expressed as a box-plot.

After metagenomic sequencing of the clinical serum EV samples was conducted, the basic characteristics of the 1727 samples selected for downstream analysis were assessed. Generally, the read count frequency of the serum samples ranged from 4- to 5-log, while OTUs hovered approximately 4-log (Fig. 1a, b). While COPD and lung cancer slightly lagged behind the control and asthma sample distribution of the total read counts, evaluation of the OTUs yielded a generally similar region of peak frequency in all groups (Fig. 1c, d). The mean read count of the total filtered samples was 55,993, while the mean number of OTUs was 10,384 (Supplementary Table 2). Sequencing yielded varying mean read counts in the control (57,252), COPD (29,441), asthma (71,328), and lung cancer (35,409) groups. However, the mean number of OTUs in the obtained serum samples was relatively similar between the control (9,589), COPD (13,039), asthma (9,736), and lung cancer (13,345) groups.

### Differential serum microbial EV alpha diversity between clinical groups

Observed OTUs, species richness estimates (Chao1 and ACE), and diversity indices (Simpson and Shannon) were used to calculate the alpha diversity of the microbial composition of COPD, lung cancer, asthma, and healthy subjects. The observed OTUs and Chao1 and ACE indices yielded the same pattern of clinical group richness values. The trend observed in ascending order of species richness was as follows: the COPD group, the healthy control group, the asthma group, and the lung cancer group (Fig. 1e). The Simpson and Shannon diversity indices also indicated similar intergroup differences in diversity, in which the COPD group had the lowest diversity with the control, asthma, and lung cancer groups, followed in ascending order of diversity (Fig. 1f).

### Taxonomic variation in serum microbial EV composition between clinical groups

**Fig. 2:**
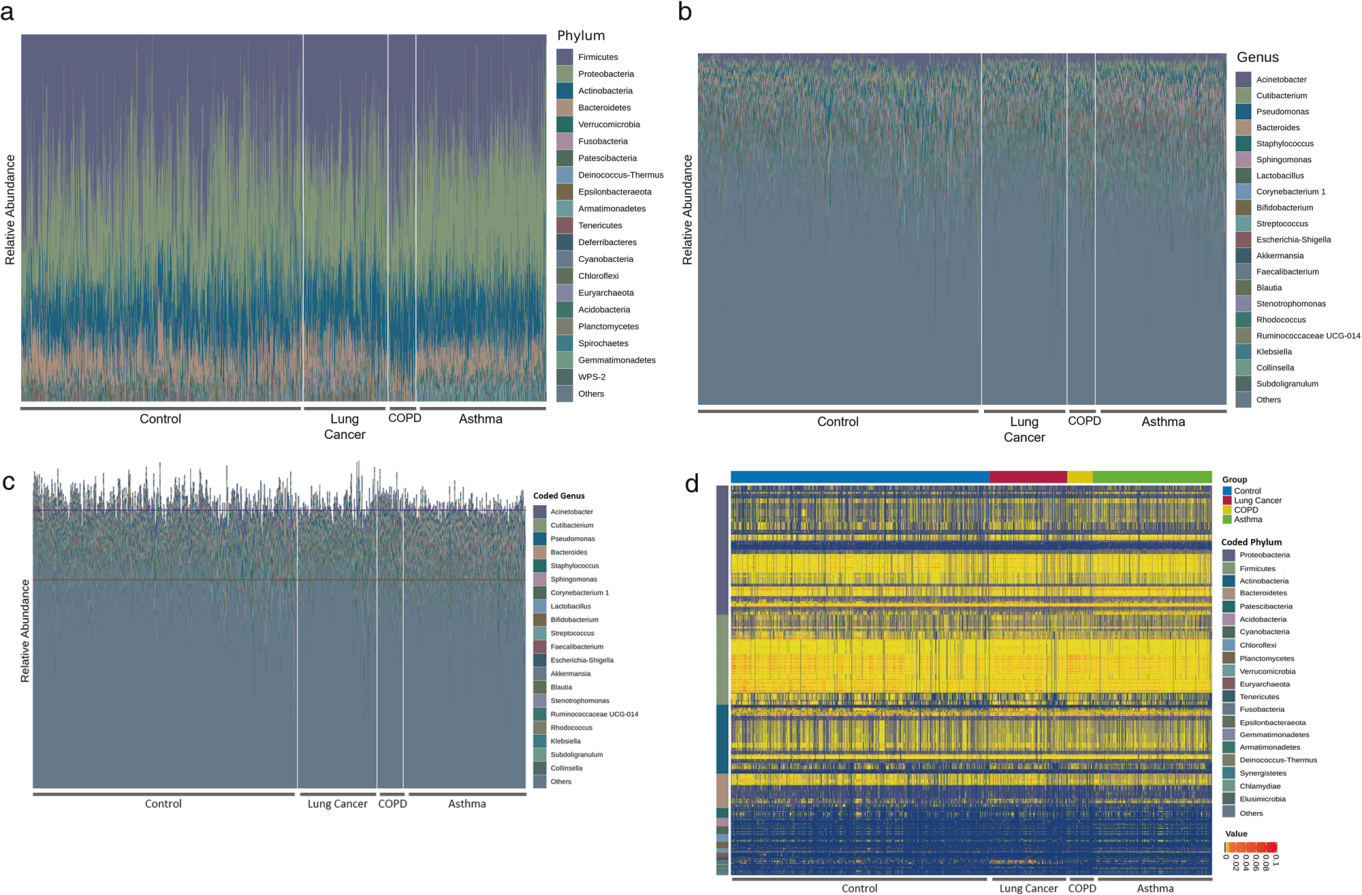
Serum microbiome compositional profiles between clinical groups. Composition of the most abundant taxa of the serum EV microbiome in healthy control, COPD, and lung cancer clinical groups at the **a** phylum and **b** genus levels was expressed as bar plots based on the relative abundance of taxa within each sample. Composition of the taxa accumulated into their respective coded taxonomies (process detailed in Methods section) was visualized by a **c** bar plot and **d** heatmap showing the relative abundance of the accumulated taxa in each clinical sample with the horizontal axis representing each clinical group and the vertical axis grouping the root phyla of the accumulated taxa.

The taxonomic composition of the microbial EVs isolated from healthy control, lung cancer, COPD, and asthma patient serum samples was determined, and the most prevalent taxa at the phylum and genus levels were plotted prior to taxonomic accumulation (Fig. 2a, b). At the phylum level, Firmicutes, Proteobacteria, Actinobacteria, and Bacteroidetes were the most abundant phyla detected in the clinical groups (Fig. 2a). A wide variety of genera were detected in the serum clinical samples, with *Acinetobacter* being the dominant genus, followed by *Cutibacterium*, *Pseudomonas*, *Bacteroides*, *Staphylococcus*, *Sphingomonas*, and *Lactobacillus* (Fig. 2b). The prominent taxa at the class, order, and family levels can be found in Supplementary Fig. 2. After taxonomic accumulation was performed as described above, the coded relative abundance of the taxa detected in each clinical sample was evaluated (Fig. 2c, d). Similar to the genus-level compositional analysis conducted prior to taxonomic accumulation, each sample possessed a wide variety of accumulated taxa, with *Acinetobacter*, *Cutibacterium*, *Pseudomonas*, *Bacteroides*, *Staphylococcus*, *Sphingomonas*, and *Corynebacterium 1* being the most predominant coded genera (Fig. 2c). Heat plot analysis of the accumulated taxa grouped by their root phylum and clinical group revealed that Proteobacteria, Firmicutes, Actinobacteria, and Bacteroidetes comprised the main portion of the coded taxa (Fig. 2d). While Proteobacteria contributed a wider variety of accumulated taxa, Firmicutes showed the most taxa with relatively high abundance in the majority of samples across all clinical groups.

### Beta diversity of clinical samples coded for taxa accumulation

**Fig. 3:**
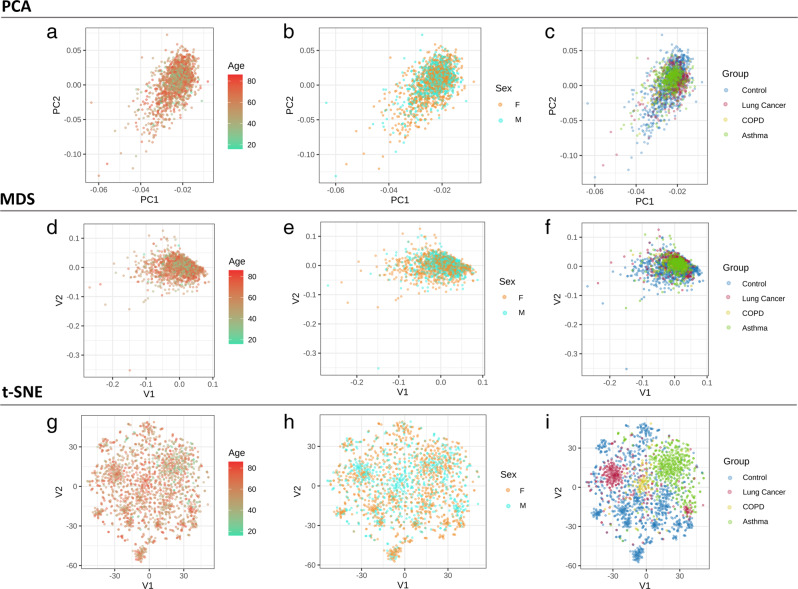
Beta diversity of clinical serum microbial EV samples. Beta diversity between groups was determined through **a**–**c** PCA, **d**–**f** MDS, and **g**–**i** T-SNE dimensional reduction methods and plotted to visualize dissimilarity of the serum samples based on age, sex, and disease group based on the accumulated taxonomic EV microbiome of each sample.

Beta diversity was measured based on PCA, MDS, and T-SNE to determine the segregation of the clinical samples based on age, sex, and clinical group (Fig. 3). The PCA and MDS methods were unable to clearly differentiate the age, sex, and clinical groups of the serum EV samples (Fig. 3a–f). However, application of the machine-learning algorithm T-SNE yielded distinct clustering between the clinical groups (Fig. 3i). The healthy control group samples were distributed throughout the V1 and V2 axes, whereas the asthma, COPD, and lung cancer samples were generally clustered separately along the V2 axis.

### Performance of serum microbial EV metagenomics-based diagnostic models

**Fig. 4:**
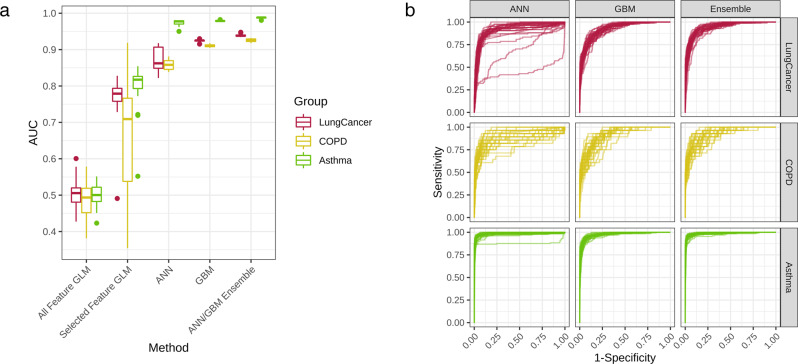
Evaluation of respiratory disease diagnostic models. **a** AUC values for 30 iterations of the lung cancer, COPD, and asthma models developed using GLM without feature selection, GLM with feature selection, ANN, GBM, and ensemble methods were visualized in box plots. **b** AUC-ROC curves of the 30 iterations were plotted against the false-positive rate and true-positive rate of the COPD, asthma, and lung cancer diagnostic models developed through ANN, GBM, and Ensemble (ANN/GBM) methods.

Diagnostic model sets were developed to determine asthma, COPD, and lung cancer risk by applying various ML methods to the coded serum EV metagenomic data. Using the five different methods (all-feature GLM, selected-feature GLM, ANN, GBM, and ANN/GBM ensemble) previously outlined, prediction models for each respiratory disease were developed. Model performance was evaluated based on the resulting receiver operating characteristic (ROC) curve of each respective methodology, and the AUC values of the test iterations for each model were plotted as a box-whisker plot (Fig. 4a). A similar pattern of model performance for each of the five models was observed in all three respiratory disease models. The all-feature GLM method showed the poorest performance, while incorporation of feature selection greatly boosted the mean AUC values of each disease model. GBM performed slightly better than ANN, with both methods outperforming the GLM-based models. The ANN/GBM ensemble yielded the highest performance in all three disease models, with mean AUC values of 0.93, 0.99, and 0.94 in the COPD, asthma, and lung cancer models, respectively (Table 1). Furthermore, there was low variation between the 30 test iterations of the ANN/GBM ensemble models with SD below 0.01 and high consistency observed between the ROC curves of the ensemble method compared to the ANN and GBM methods in all three respiratory models (Fig. 4b).

**Table 1:** Respiratory disease diagnostic model performance across methodologies.

Model	Method	Mean AUC	SD of AUC
COPD	All-feature GLM	0.49	0.054
	Selected-feature GLM	0.66	0.158
	ANN	0.86	0.012
	GBM	0.91	0.003
	ANN/GBM ensemble	0.93	0.004
Asthma	All-feature GLM	0.50	0.031
	Selected-feature GLM	0.80	0.057
	ANN	0.97	0.007
	GBM	0.98	0.001
	ANN/GBM ensemble	0.99	0.002
Lung cancer	All-feature GLM	0.50	0.040
	Selected-feature GLM	0.77	0.057
	ANN	0.87	0.030
	GBM	0.92	0.003
	ANN/GBM ensemble	0.94	0.004

*AUC* area under the curve, *SD* standard deviation, *COPD* chronic obstructive pulmonary disease, *GLM* general linearized model, *ANN* artificial neural network; *GBM* gradient boosting machine.

### Feature importance variation between respiratory disease models

**Fig. 5:**
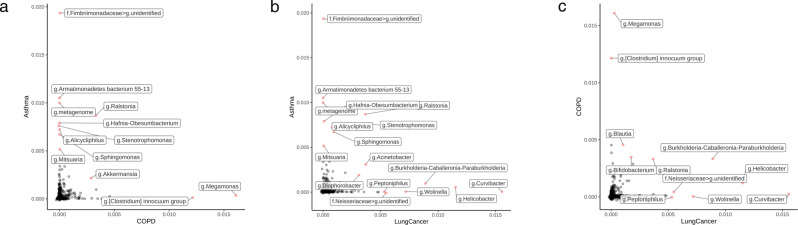
Relatively important features of the respiratory disease models. The feature importance of the diagnostic models was assessed based on permutation feature importance analysis of the coded serum microbial EV taxa. Individual features of highest importance were compared between the **a** asthma and COPD models, **b** asthma and lung cancer models, and **c** COPD and lung cancer models.

Permutation feature importance analysis was subsequently performed on the three respiratory disease models to determine which coded serum microbial EV taxa had the most impact on disease prediction outcomes. In asthma and lung cancer, the phylum Proteobacteria accounted for the most features important to the model, while in COPD, Firmicutes was the most prevalent phylum for feature importance (Supplementary Table 3). When comparing feature importance between asthma and COPD, several features identified to be a part of the *Fimbriimonadaceae* family, represented solely by the genus *Fimbriimonas*, were highly associated with asthma, while *Megamonas* was most associated with COPD. Several features were mildly associated with both asthma and COPD, including *Ralstonia* (Fig. 5a). *Curvibacter* and *Helicobacter* were the most important features in the lung cancer model, while several features of slight to moderate importance were shared between the asthma and lung cancer models, including *Stenotrophomonas*, *Acinetobacter*, and *Ralstonia* (Fig. 5b). Finally, a genus identified as *Burkholderia-Caballeronia-Paraburkholderia* and *Ralstonia* shared mild to moderate importance between the COPD and lung cancer models (Fig. 5c).

### Modulation of HFD-induced respiratory disease risk by dietary supplements

**Fig. 6:**
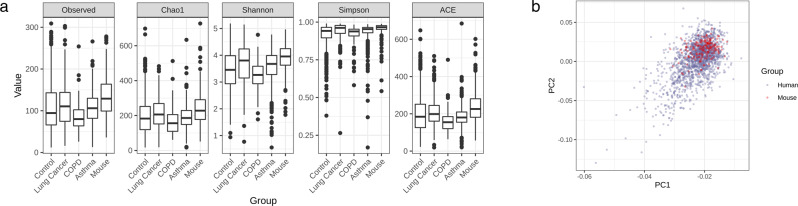
Comparison of human and mouse serum microbial EV sample diversity. **a** Alpha diversity was calculated for the control, lung cancer, COPD, and asthma patient sample groups in addition to the mouse samples obtained through the in vivo dietary study. **b** PCoA analysis of mouse samples and human clinical samples used for respiratory disease diagnostic model development was conducted and plotted.

**Fig. 7:**
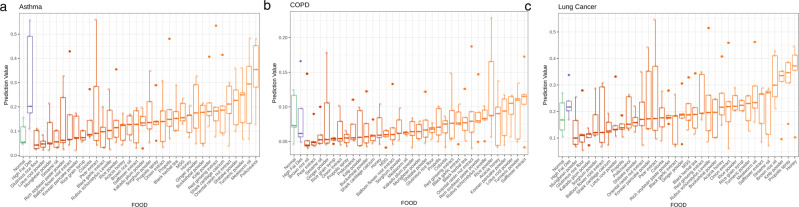
Effect of diet on in vivo respiratory disease risk. The prediction values for **a** asthma, **b** lung cancer, and **c** COPD were plotted as box-whisker plots for mice fed normal chow or a high-fat diet (HFD) followed by the HFD groups supplemented with different health foods in ascending order of disease prediction values (*n* = 5).

An in vivo animal study was conducted to determine the ability of various dietary proteins, vegetables, lipids, and grains to modify HFD-associated respiratory disease risk in mice. Alpha diversity was analyzed and compared between the four human clinical groups (healthy control, lung cancer, COPD, and asthma) and samples collected from the mice used in the in vivo dietary study (Fig. 6a). All matrices used to calculate alpha diversity, observed OTUs, Chao1, Shannon, Simpson, and ACE, demonstrated that the mouse samples had slightly higher diversity than the clinical samples. The UniFrac distance between the 180 mouse samples and the 1727 clinical samples used in the development of the COPD, asthma, and lung cancer diagnostic models was assessed. PCoA analysis showed a slightly tighter clustering of most mouse samples within the broader distribution of human samples (Fig. 6b). The ANN/GBM ensemble respiratory disease models were applied to the serum samples obtained from the 36 dietary groups, and the resulting disease model prediction values were plotted (Fig. 7). HFD influenced asthma prediction the most, with a mean prediction value of 0.31 compared to 0.09 in the NCD group. Meanwhile, application of the lung cancer model to HFD-fed mice yielded a mean prediction value of 0.23 compared to 0.18 in the NCD group. COPD risk was impacted the least by a HFD, with a mean prediction value of 0.09 in both the HFD and NCD groups.

Comparison of disease prediction values between the dietary groups revealed that the asthma model yielded the highest number of foods that minimized HFD-associated disease risk, including glutinous rice flour, lotus root powder, mungbean powder, and sesame oil (Fig. 7a). Conversely, foods such as policosanol and mealworm oil increased asthma prediction values. HFD-associated lung cancer prediction values were also lowered by multiple food supplements, such as mungbean powder, glutinous rice flour, and Kakadu plum powder (Fig. 7b). However, honey and propolis spray drastically boosted lung cancer prediction values. COPD prediction values were not dramatically altered by diet (Fig. 7c). Several food items, including brown rice oil, pear extract, and sesame oil, lowered the COPD prediction values below those of the NCD and HFD groups, while safflower extract slightly increased COPD risk.

## Discussion

Serum microbial EVs have potential as diagnostic markers for a wide range of diseases, as they systemically interact with our immune and organ systems. Here, we determined the serum EV microbiome composition of healthy subjects, COPD patients, asthma patients, and lung cancer patients. After coding serum EV metagenomic data with a novel taxonomic hierarchal clustering method, we developed powerful asthma, COPD, and lung cancer predictive diagnostic models using an ANN/GBM ensemble method. The results of this study highlight the diagnostic capability of serum microbial EVs as features for respiratory disease prediction and serve as a steppingstone for the development of a novel, accurate and noninvasive diagnostic method for chronic diseases lacking a diagnostic gold standard.

Although researchers have previously used serum as a biomarker source for asthma, COPD, and lung cancer, this is the first study to use a serum microbial EV metagenomic approach for all three respiratory diseases. Standard lung cancer diagnostic methods are rarely suitable for early detection, tend to be highly invasive, and yield low sensitivity through noninvasive methods^20^. Systemic reviews of asthma and COPD diagnostic methods showed that tests commonly used in primary care, such as handheld flow meters and hyperresponsiveness testing, have limited sensitivity and specificity^21,22^. Furthermore, asthma and COPD can be difficult to differentiate due to symptom similarity and can lead to fatality without proper treatment, particularly in the case of COPD^23^. Therefore, recent efforts have focused on using biomarkers for higher accuracy and sensitivity of respiratory disease prediction. Several other serum biomarkers previously targeted for COPD, asthma and lung cancer diagnosis include metabolic profiling^24,25^, endothelial microparticles^26^ and serum miRNA profiles^27,28^. Differential DNA methylation profiles have also been utilized to develop lung cancer and COPD diagnostic models with high accuracy in lung cancer but lower strength in COPD^29^. Serum immunoglobulin E (IgE) levels have been used for asthma diagnosis with varied success^30,31^.

Several reliable ML classification methodologies were tested in this study to determine an optimal method for diagnostic model development using microbial EV metagenomic data as model features. Microbiome studies have exploded in popularity over the past decade, resulting in large amounts of data, a multitude of conflicting findings, and the need for a higher-level analytical technique for the classification of metagenomic data. As a result, ML algorithms have been embraced as a powerful tool for interpretation of the complex big data output of microbiome profiling^32,33^. To overcome several challenges in microbiome analysis, we also developed and applied a novel data-processing method. Microbiome count data typically have a high abundance of zero values, which creates a bias that must be corrected prior to use as model input features. Additionally, taxonomic assignment is often based on public databases in which many entries are loosely specified at the genus level^34^. Rather than removal of all imprecisely labeled taxa, resulting in diminished data resolution, we employed a novel method in which taxonomic hierarchal values of a given OTU were clustered into a single coded value. Through this method, we aimed to address these biases and improve the rationale of our diagnostic models. While further validation is necessary, the consistently high diagnostic strength shown through multiple iterations of the models trained with coded metagenomic data supports the merit of our taxonomic hierarchal coding method.

This study was the first systemic microbial EV metagenomic approach to COPD, asthma, and lung cancer diagnosis and yielded many microbiome features of respiratory disease that diverged from those previously reported. Preceding studies that targeted the microbiome for respiratory disease biomarkers primarily assessed the lung, airway, and oral microbiota, with each area yielding unique microbiota compositions. Here, feature importance analysis conducted for each respiratory disease model revealed that genera derived from Firmicutes influenced the distinction of COPD and healthy systemic microbiome activity the most. A review of previous COPD microbiome studies showed that while Firmicutes prevalence is often increased in COPD patients across a variety of clinical samples, including lung tissue and endotracheal aspirates, there is less consensus on how this prevalence affects the overall diversity of COPD patients^35^. Increased Firmicutes abundance is often associated with overall increased diversity due to the abundance of human microbiome-associated genera represented by the phylum. However, we found that the COPD group was the only respiratory disease group to be less diverse than the healthy control group.

Conversely, Proteobacteria emerged as the dominant phylum in both the asthma and lung cancer disease groups. Increased Proteobacteria prevalence in the airway has been a hallmark of both cancerous and asthmatic lung microbiome studies^36,37^. At the genus level, *Stenotrophomonas*, a member of the Proteobacteria phylum, was determined to be important to both the asthma and lung cancer models. *Stenotrophomonas maltophilia*, the most common *Stenotrophomonas* spp., has been previously identified to have pathogenic potential in the lungs. The species can activate host TLR5-mediated pro-inflammatory responses through flagellin, which has also been detected in the EVs of flagellated bacteria^36,38^. *Helicobacter*, a genus containing several well-known pathogens, including the carcinogen *Helicobacter pylori*, was also an important feature of the lung cancer model. While *Helicobacter* spp. such as *H. plyori* have been previously associated with lung cancer, the most important feature in the model, *Curvibacter*, has been linked to COPD but not lung cancer^39^. Although *Megamonas* was the genus with the most weight in the COPD group, to our knowledge, there have been no previous reports of its association with COPD or other respiratory diseases. Similarly, an unidentified genus of the *Fimbriimonadaceae* family was shown to be the most important feature of asthma; however, no publications were found to mention *Fimbriimonas*, the sole genus of the family, in relation to respiratory disease. Therefore, further investigation into the role of *Fimbriimonas*, *Curvibacter*, and *Megamonas* activity in asthma, COPD, and lung cancer, respectively, may be warranted.

Consumption of Western diets, such as a HFD, has been associated with an increased risk of various chronic diseases. It has been known for decades that diets high in fat and processed foods are associated with higher lung cancer risk^40,41^. Furthermore, HFD has been directly shown to promote lung cancer progression in a mouse Lewis lung carcinoma (LLC) allograft model through the modulation of cell signaling pathways resulting in increased JAK, NF-κB, and STAT3 levels as well as increased oxidative stress^42^. Aside from oncologic respiratory consequences, HFD has also been associated with asthma incidence. In a previous population study, it was found that, in men, the intake of a HFD was more highly associated with asthma than a smoking history was^43^. While a HFD has been closely associated with lung cancer and asthma, the relationship between HFD and COPD is less clear. A HFD has been suggested to be beneficial to COPD patients due to the impact of fat on their respiratory quotient (RQ) by lower production of CO_2_ following fat metabolism. In a 1993 study, it was reported that a HFD yielded lower values of carbon dioxide production, oxygen consumption and RQ than a diet high in carbohydrates in COPD patients^44^.

Interestingly, several key results of the in vivo dietary assessment conducted in this study were in general agreement with the literature. Application of the human microbial EV metagenomic disease models developed in this study to mice fed a HFD demonstrated increased risk for asthma and lung cancer while exerting a minimal effect on COPD risk. Furthermore, the ability of various food items to modulate HFD-associated disease risk was drastically lower in the COPD model than in the asthma and lung cancer models. Mungbean powder, Kakadu plum powder, and lotus root powder extract showed particular promise as food items capable of offsetting HFD-associated respiratory disease risk. Mungbean contains various bioactive components, and numerous studies have demonstrated its ability to reduce pro-inflammatory responses in vitro and in vivo^45^. Recently, mungbean supplementation was shown to normalize the gut microbiota of obese mice and reduce obesity-associated metabolic disorder and symptoms^46^. *Terminalia ferdinandiana* (Kakadu plum) contains antioxidant phytochemicals and has demonstrated antimicrobial efficacy against various foodborne and medical pathogens^47^. In this study, lotus root powder showed an especially potent effect against asthma disease risk and is known to have anti-inflammatory properties. Lotus root powder reduces serum inflammatory markers such as total IgE and leukotriene B4, both of which play a role in airway hyperresponsiveness and pro-inflammatory responses^48,49^. Altogether, the results of the in vivo dietary assessment offer preliminary validation of our serum microbial EV-based disease risk models and reveal foods that may reduce the HFD-associated respiratory disease risk.

In conclusion, the results of this study highlight the utility of serum microbial EVs as potent features for asthma, COPD, and lung cancer prediction. While the models yielded high AUC values, further study is necessary to clinically validate the models in larger cohorts at specific stages of disease progression and with more control samples. Future studies should include a wider range of patients in terms of age and geographic region as well as their medication and smoking history to enhance the strength and applicability of the diagnostic models. Clinical validation must also be conducted to verify dietary supplements capable of offsetting the HFD-associated lung cancer or asthma risk.

## Supplementary information

Supplementary information
