# Supplementary material for: Machine-learning algorithms for asthma, COPD, and lung cancer risk assessment using circulating microbial extracellular vesicle data and their application to assess dietary effects
Source: Exp Mol Med. 2022 Sep 30;54(9):1586–95. doi: 10.1038/s12276-022-00846-5 (PMC9534896; doi:10.1038/s12276-022-00846-5)
Supplement: Supplementary file 1 — Supplementary information [file 12276_2022_846_MOESM1_ESM.pdf]

## Supplementary Figure Legends

### Supplementary Fig. 1. Correlation between age, sex, and serum microbiome

Pearson's correlation was evaluated at principal components 1 and 2 (PC1 and PC2) to determine if there was significant correlation between the **a)** age or **b)** sex of clinical subjects and their serum microbiome profiles. Significance was established at $p \leq 0.05$.

### Supplementary Fig. 2. Serum microbiome compositional profiles between clinical groups (class, order, and family levels)

Composition of the most abundant taxa of the serum EV microbiome in healthy control, COPD, and lung cancer clinical groups at the **a)** class, **b)** order, and **c)** family levels was expressed as bar plots based on the relative abundance of taxa within each sample.

**Supplementary Fig. 1**

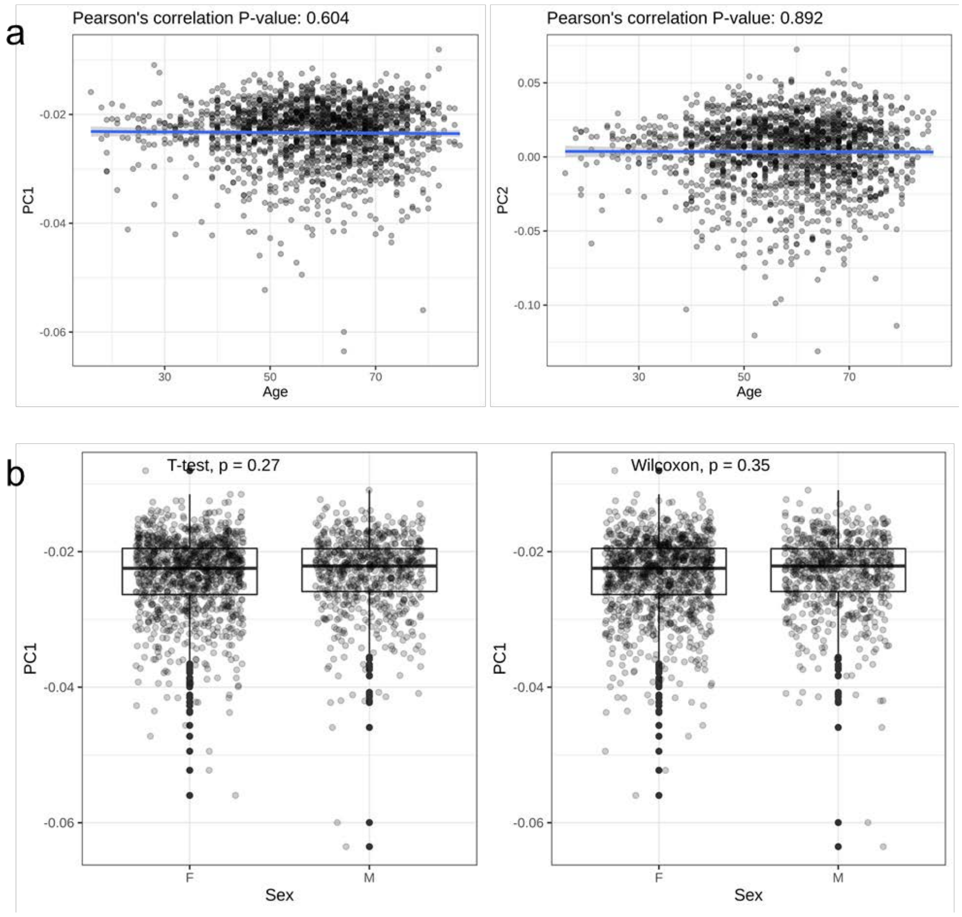

**Supplementary Fig. 2**

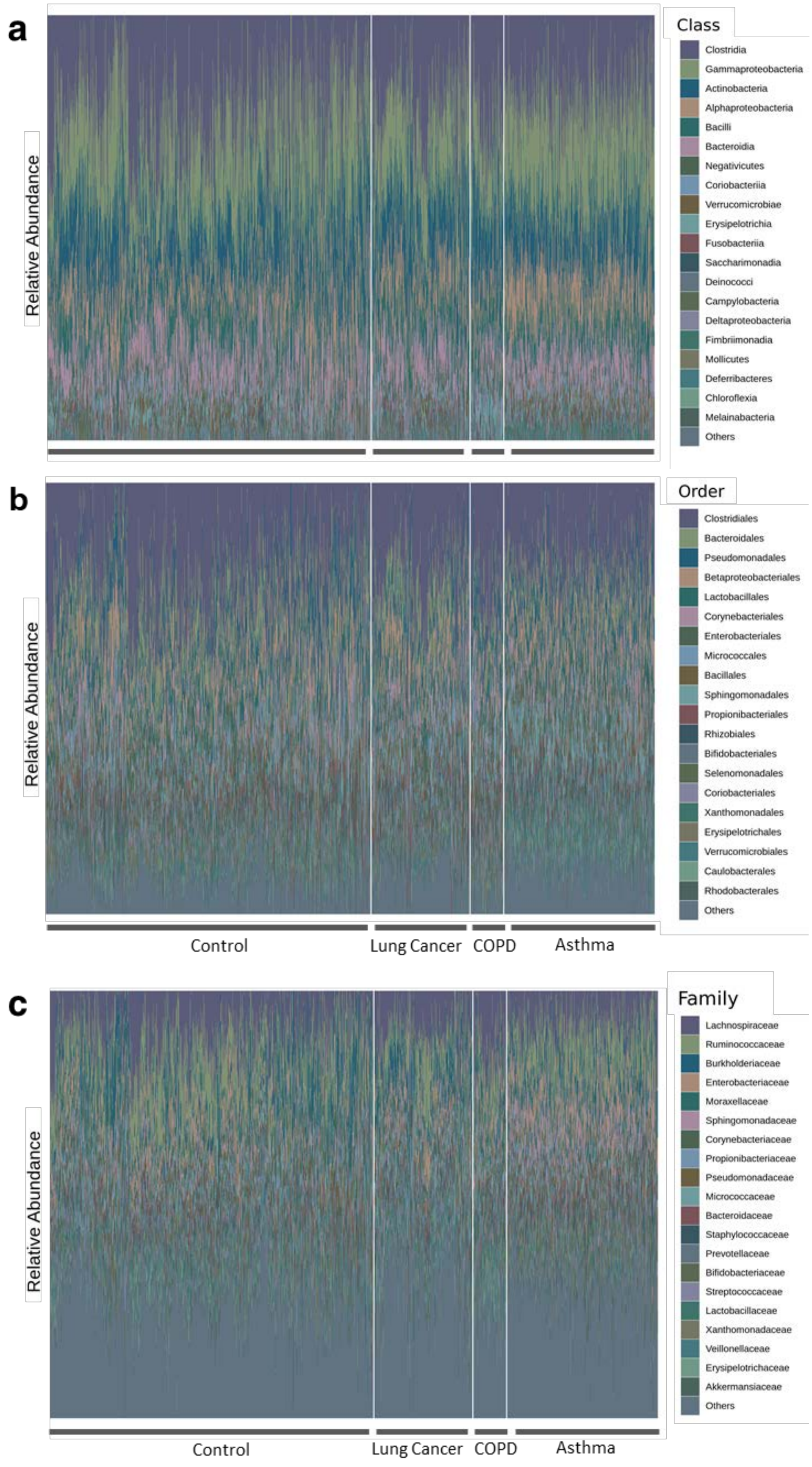

## Supplementary Tables

**Supplementary Table 1.** Basic subject clinical characteristics

| Group | Institution | No. | Sex (M/F) | Age (mean [SD]) |
|---|---|---|---|---|
| Control | Inje University Haeundae Hospital | 928 | 0.35 | 58.78 [10.68] |
| COPD | Konkuk University Medical Center | 92 | 22.00 | 69.68 [7.33] |
| Asthma | Asan Medical Center | 428 | 0.69 | 51.53 [15.59] |
| Lung cancer | Samsung Medical Center | 251 | 1.68 | 62.72 [10.67] |
| | Dankook University Hospital | 28 | | |

COPD: Chronic Obstructive Pulmonary Disease; SD: Standard Deviation.

**Supplementary Table 2.** Summary of raw serum microbial EV sample read counts and profiled OTU counts

| | Read Count | | | OTU | | |
|---|---|---|---|---|---|---|
| Group | Mean | Median | SD | Mean | Median | SD |
| Control | 57,252 | 42,485 | 40,749 | 9,589 | 8,457 | 6,929 |
| COPD | 29,441 | 25,908 | 13,283 | 13,039 | 12,012 | 5,354 |
| Asthma | 71,328 | 64,823 | 33,806 | 9,736 | 7,704 | 7,750 |
| Lung cancer | 35,409 | 28,359 | 20,597 | 13,345 | 11,022 | 8,249 |
| Total | 55,993 | 44,444 | 37,745 | 10,384 | 9,145 | 7,433 |

OTU: Operational Taxonomic Unit; COPD: Chronic Obstructive Pulmonary Disease; SD: Standard Deviation.

**Supplementary Table 3**. Assessment of important serum microbial EV features in respiratory disease models

| Model | Phylum | Weight | No. of Features | Feature Weight Range |
|---|---|---|---|---|
| COPD | Firmicutes | 0.092 | 326 | -2.15E-04 ~ 1.82E-02 |
| | Actinobacteria | 0.037 | 250 | -1.64E-04 ~ 2.72E-03 |
| | Verrucomicrobia | 0.015 | 17 | 5.82E-04 ~ 2.16E-03 |
| | Proteobacteria | 0.015 | 469 | -2.05E-04 ~ 4.07E-03 |
| | Bacteroidetes | 0.006 | 125 | -1.44E-04 ~ 1.09E-03 |
| | Epsilonbacteraeota | 0.002 | 6 | 1.05E-05 ~ 1.61E-03 |
| | Acidobacteria | 0.001 | 28 | -3.06E-05 ~ 4.66E-04 |
| Asthma | Proteobacteria | 0.123 | 469 | -1.62E-04 ~ 9.60E-03 |
| | Armatimonadetes | 0.034 | 3 | 9.64E-04 ~ 2.09E-02 |
| | Bacteroidetes | 0.022 | 125 | -4.78E-05 ~ 3.33E-03 |
| | Actinobacteria | 0.016 | 250 | -1.25E-04 ~ 3.31E-03 |
| | Firmicutes | 0.012 | 326 | -1.49E-04 ~ 2.22E-03 |
| | Verrucomicrobia | 0.009 | 17 | 6.73E-05 ~ 1.71E-03 |
| | Cyanobacteria | 0.002 | 31 | -3.84E-06 ~ 5.71E-04 |
| Lung cancer | Proteobacteria | 0.090 | 469 | -1.28E-04 ~ 1.59E-02 |
| | Firmicutes | 0.062 | 326 | -1.01E-04 ~ 5.58E-03 |
| | Epsilonbacteraeota | 0.031 | 6 | 7.70E-04 ~ 1.13E-02 |
| | Actinobacteria | 0.020 | 250 | -1.61E-04 ~ 1.91E-03 |
| | Bacteroidetes | 0.009 | 125 | -7.15E-05 ~ 3.19E-03 |
| | Deinococcus-Thermus | 0.008 | 4 | 1.70E-04 ~ 4.87E-03 |
| | Verrucomicrobia | 0.006 | 17 | 9.70E-06 ~ 1.90E-03 |
| | Tenericutes | 0.003 | 12 | -3.36E-05 ~ 1.99E-03 |
| | Fusobacteria | 0.002 | 7 | 1.12E-04 ~ 5.19E-04 |
